# Alzheimer disease may compromise patients' ability for expectancy-based pain modulation. Now what?

**DOI:** 10.1097/j.pain.0000000000003036

**Published:** 2023-09-13

**Authors:** Katja Wiech, Ulrike Bingel

**Affiliations:** a Wellcome Centre for Integrative Neuroimaging (WIN), Nuffield Department of Clinical Neurosciences, University of Oxford, Oxford, United Kingdom; b Department of Neurology, Center for Translational Neuro- and Behavioural Sciences, University Hospital Essen, Essen, Germany

The observation that positive treatment expectations can improve treatment outcome by engaging powerful pain modulatory mechanisms in the brain has broadened the way we conceptualise and treat pain.^2^ Moreover, increased research efforts in this field have raised hopes that we can harness these mechanisms in therapeutic approaches. But what about those who only have limited access to the body's own “pharmacy”? A study by Matthiesen et al.^5^ suggests that patients with Alzheimer disease (AD) may belong to this group.

Using a within-subject design, Matthiesen et al. compared the effects of open and hidden application of a local anesthetic (lidocaine); capsaicin, which increases sensitivity to thermal stimuli; or a cream containing no active ingredient in patients with AD and healthy volunteers. A greater analgesic effect after open compared with hidden application of lidocaine would suggest that analgesia was caused by a combination of pharmacologic action and a placebo effect. However, in contrast to the control group that showed stronger pain modulation after open application, pain intensity ratings of patients with AD were not significantly different in the 2 conditions. This finding confirms a previous report of a reduced placebo effect in this group of patients.^1^

The lack of an expectancy-based pain modulation in patients with AD may be due to a variety of factors—and understanding their relative importance will be critical to drawing the right conclusions from findings such as those reported by Matthiesen et al. Contemporary theories suggest that perception is the result of sensory information and expectations that we generate based on previous information (refer to, eg, Ref. 6 for an overview). Dementia has been postulated to affect the delicate process that integrates these 2 components.^4^ The impact of expectations also depends on the individual's capacity to generate and maintain them, and to use their influence on pain effectively by activating of top–down pain modulatory processes. Neurodegeneration and functional changes in key brain regions such as the dorsolateral prefrontal cortex,^3^ which orchestrates top–down pain modulation, may damage the necessary “hardware” to the extent that sufficient pain modulation is prevented. Patients with AD may also no longer be able to retain the information needed to produce a placebo effect. As a result, the lack of strong expectations could lead to an unfiltered influx of nociceptive information and an overemphasis on sensory evidence in the perceptual process.

An important observation in the study by Matthiesen et al. reinforces the notion of a disconnect between patients' expectations and subsequent perception. The authors asked participants immediately after treatment application how painful they expect the subsequently applied thermal stimuli to be. Their results show stronger expectations of pain relief in the open than the hidden application condition, suggesting that patients were able to generate positive treatment expectations based on the information they had received. However, the finding that this had no significant effect on pain perception suggests that the positive expectation had either dissipated by the time the stimulation was applied or that it was not strong enough to influence perception. The fact that patients also showed no significant nocebo effect (defined as higher pain ratings during open than hidden application of capsaicin controlled for natural history) should only be of little comfort and suggests that the observed deficit is not specific to positive expectations.

It should be noted that the study by Matthiesen et al. is based on a relatively small sample size and focused on patients with mild to moderate AD (mean Mini-Mental State Examination [MMSE] score of 23), who may still be able to compensate for their loss of cognitive function. Longitudinal studies to track changes in patients' ability to produce expectancy-based pain modulation as well as larger scale observational and experimental studies with direct comparisons between patients with AD at different stages of the disease and relevant control groups are urgently needed. Furthermore, other neurological conditions, such as multiple sclerosis, stroke, or Parkinson disease, may cause similar deficits which should be investigated using carefully selected assessment techniques that take into account patients' varying ability levels. Matthiesen et al. are to be commended for their efforts to adapt their data collection methods to the challenges of impaired cognitive function (eg, using a numerical rating scale rather than the more difficult-to-use visual analogue scale).

If confirmed in larger scale follow-up studies, the findings of Matthiesen et al. would have far-reaching implications. However, rather than eliminating the placebo control condition in clinical trials in patients with AD (as some have argued and the authors rightly criticized), we propose exploring more creative approaches to preserving the capacity for expectancy-based pain modulation while adapting to the challenges that progressing dementia may present. For instance, in addition to providing information about the intervention only at the start of treatment, implementing periodic reminders that engage multiple senses—such as pairing medication with a distinct taste or scent (eg, Ref. 7)—could help reinforce the beneficial effects of treatment. By evoking sensory associations, these interventions offer an immersive experience that could sustain positive expectations even in the face of memory problems and cognitive decline. Moreover, ritualised application techniques can tap into the well-established benefits of conditioned responses. Incorporating consistent routines and procedural elements into treatment administration may not only enhance the placebo effect but also add a sense of familiarity and predictability for patients. Such approaches may be of paramount importance particularly for those in more advanced stages of the disease who often face comorbidities and complications that limit their pharmacologic options. However, impaired top–down regulation of pain may also require adjustment of the type and dosage of analgesics (if feasible) in addition to the use of behavioral techniques. In patients with dementia, access to effective treatment is frequently impeded, leaving them undertreated and vulnerable. By harnessing the potential of the placebo effect through tailored interventions and treatment modifications that take into account disease-related changes in endogenous pain modulation, we could bridge the gap in care and ensure that this group receives the support they need.

## Conflict of interest statement

The authors have no conflicts of interest to declare.
